# Effect of Genistein Supplementation on Exercise-Induced Inflammation and Oxidative Stress in Mice Liver and Skeletal Muscle

**DOI:** 10.3390/medicina57101028

**Published:** 2021-09-27

**Authors:** Cong Wu, Siyi Zhou, Sihui Ma, Katsuhiko Suzuki

**Affiliations:** 1 Graduate School of Sport Sciences, Waseda University, Tokorozawa 359-1192, Japan; wucong86@foxmail.com (C.W.); zhousiyi941@toki.waseda.jp (S.Z.); 2 Faculty of Sport Sciences, Waseda University, Tokorozawa 359-1192, Japan; 3 Japan Society for the Promotion of Sciences, Chiyoda-ku, Tokyo 102-0083, Japan

**Keywords:** genistein, inflammation, oxidative stress, pro-oxidant, exhaustive exercise

## Abstract

*Background and objectives*: The purpose of this study was to investigate the influences of oral high-dose genistein (GE) administration on exercise-induced oxidative stress, inflammatory response and tissue damage. *Materials and Methods*: Thirty-two mice were randomly divided into control group (Con; sedentary/0.5% CMC-Na), GE administrated group (GE; sedentary/GE dosed), exercise group (Ex; exercise/0.5% CMC-Na), or GE administrated plus exercise group (GE + Ex; exercise/GE dosed), mice in the GE and GE + Ex group were given GE orally at the dose of 200 mg/kg weight. *Results*: Plasma aspartate aminotransferase (AST), alanine aminotransferase (ALT) levels, liver interleukin (IL)-6, IL-1β, superoxide dismutase 1 (*SOD1*), catalase (*CAT*), hemeoxygenase-1 (*HO-1*) gene expression levels and skeletal muscle IL-6, nuclear factor erythroid 2-related factor (*Nrf2*), and *HO-1* gene expression levels increased immediately after exhaustive exercise. GE supplementation increased liver protein carbonyl concentrations. On the other hand, GE supplementation significantly decreased *SOD1*, *CAT* gene expression levels in the liver and *Nrf2*, and *HO-1* gene expression levels in the skeletal muscles. *Conclusions*: Acute exercise induced organ damage, inflammation, and oxidative stress in skeletal muscles and the liver. However, a single dose of GE supplementation before exercise did not lead to favorable antioxidant and anti-inflammatory effects in this study.

## 1. Introduction

Oxidative stress is defined as a kind of imbalance caused by electronically excited states and reactive oxygen species (ROS) in organisms [1]. When the production rate of ROS exceeds the system’s detoxification and repair capacity, oxidative stress can result in cellular damage [1]. ROS consists of various derivatives of oxygen, for instance, superoxide anion radical (O_2_^▪−^), hydrogen peroxide (H_2_O_2_), hydroxyl radical (HO^▪^), and singlet oxygen (^1^O_2_) [2].

Physical exercise is widely considered as an effective way to maintain health status [3,4]. However, strenuous exercise may lead to an increase in the production of ROS, whereas the source, function, and response of ROS during exercise remain disputed topics. According to Suzuki et al. and Radak et al. [5,6], when the intensity of exercise is moderate, organisms can adapt to the mild burst of ROS; this kind of adaptation is called hormesis. However, adaptation will not occur with “too heavy” exercise bouts or intensity. Exhaustive exercise will lead to the cellular production of ROS and exceed the capacity of the antioxidant defense system. When the homeostatic condition of redox (oxidation-reduction) balance breaks, ROS reacts with cellular components, including DNA, lipids, and proteins, and can lead to cellular damage, which can be called short-term oxidative stress. Furthermore, this kind of episodic increase in ROS production may induce muscle damage, oxidative stress, and inflammation simultaneously [7]. When free radical damage is caused by high-intensity exercise, the pro-inflammatory mediators, such as interleukin (IL)-6, IL-1β, tumor necrosis factor (TNF)-α, and nitric oxide (NO) will be induced afterwards. There are plenty of studies indicating that the infiltrated neutrophils and macrophages in active tissues may take part in the release of pro-inflammatory cytokines. On the other hand, some researchers also reported that when muscle damage occurs, neutrophils may deteriorate damage by releasing ROS [8]. The increasing cytokines and ROS can be the key drivers of inflammation and the followed myofiber membrane lysis [9].

Some studies have investigated the possibility of protecting the body from exhaustive exercise-induced muscle damage, oxidative stress, and inflammation by using antioxidant supplements. These kinds of protective effects indicate that the administration of antioxidant supplements can be a reasonable strategy to enhance recovery and performance [10,11,12].

Flavonoids are plant phenolic compounds that have different kinds of medicinal properties. They are widely distributed in almost every plant, especially in fruits and vegetables. Isoflavonoids are one of the subgroups of flavonoids, widely used as a kind of potent antioxidant supplement. Among the isoflavonoids family, genistein (4′,5,7-trihydroxyisoflavone, GE) is one of the most studied isoflavonoids, which is rich in soy-derived foods due to its estrogenic functions and antioxidative, anti-inflammatory activities. GE is usually ingested by humans, mainly as the glycoside form of GE, which is called genistin. After the process of bacteria hydrolysis, genistin is absorbed in the form of GE [13].

**Figure 1:**
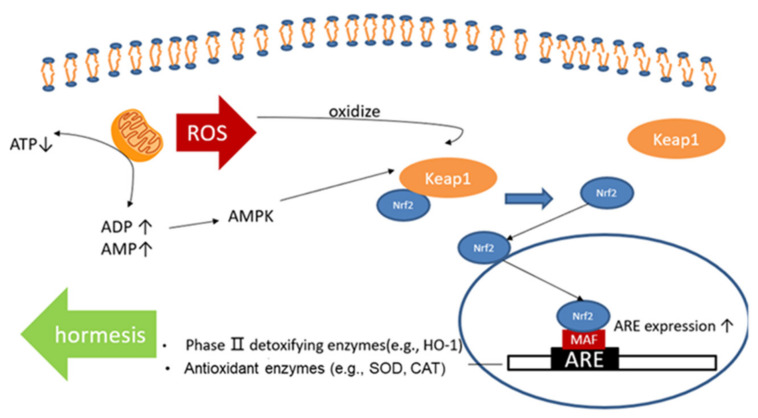
Nrf2 signaling. Nrf2 is activated by ROS. ROS, reactive oxygen species; Keap1, Kelch-like ECH-associated protein 1; Nrf2, nuclear factor erythroid 2-related factor; HO-1, hemeoxygenase-1; MAF, musculoaponeurotic fibrosarcoma; ARE, antioxidant response element; HO-1, hemeoxygenase-1; SOD, superoxide dismutase; CAT, catalase.

As the major upstream stimulatory factor of various phase II detoxification and antioxidant enzymes, nuclear factor erythroid 2-related factor (Nrf2) is fully discussed in the previous study [14]. There are two states of Nrf2 in the cellular environment. Nrf2 is generally conjoined with Kelch-like ECH-associated protein 1 (Keap1), a negative regulator. In that case, Nrf2 is settled in the cytoplasm, facing further ubiquitination and subsequent proteolysis [15]. However, the conformational state of Keap1 can be altered by the oxidation activity of ROS production, which will lead to detachment. Once detached from Keap1, Nrf2 is translocated into the nucleus, where it has the ability to heterodimerize with the MAF protein and bind to the expression of antioxidant response elements (AREs) [16]. A high level of oxidative stress, which is caused by physical exercise, can upgrade the release of Nrf2 and increase expression of ARE, which includes hemeoxygenase-1 (HO-1), superoxide dismutase (SOD), and catalase (CAT) (Figure 1) [17].

Although there are still plenty of studies investigating the effects of GE treatment on preventing exercise-induced oxidative stress and organ damage, most previous studies used relatively long-terms and relatively small doses during the administration of GE and the conclusions are mixed, partially resulting from the different dosages and the inconsistent administration period. In order to investigate whether a single dose of GE administration is effective at preventing the unwanted situations during exhaustive exercise, in this study, we used a relatively high and single dose of GE as a pre-treatment, in order to investigate the influences of GE on exercise-induced oxidative stress, inflammatory response, and muscle damage.

## 2. Materials and Methods

### 2.1. Animals

Thirty-two male C57BL/6J mice (8 weeks old) were purchased from Takasugi experimental animals supply (Kasukabe, Japan). Four mice were housed in one cage (27 × 17 × 13 cm) in a controlled environment under a light-dark cycle (lights on at 9:00 and off at 21:00). The experimental procedures followed the Guiding Principles for the Care and Use of Animals in the Waseda University Institutional Animal Care and Use Committee in the university. Thirty-two mice were randomly divided into four groups (*n* = 8): control group (Con; sedentary/0.5% CMC-Na), GE administrated group (GE; sedentary/GE dosed), exercise group (Ex; exercise/0.5% CMC-Na), or GE administrated plus exercise group (GE + Ex; exercise/GE dosed).

### 2.2. Experimental Protocol

All mice were trained with treadmill at the speed of 15 m/min for 10 min one week before exhaustive exercise in order to be familiarized with treadmill running. One hour before the exhaustive exercise procedure, mice in the GE and GE + Ex group were given GE (FUJIFILM Wako Pure Chemical Co., Japan. Item no. 079-05533) orally at the dose of 200 mg/kg weight, which suspended in 0.5% CMC-Na solution. The amount of administration was chosen based on previous reports [18]. Then, mice in the Ex and GE + Ex groups performed the exercise protocol (running at the speed of 10 m/min, 15 m/min, 20 m/min, for 15 min, respectively, at a 7% grade, keeping the speed of 24 m/min until exhaustion) on treadmill (Natsume, Kyoto, Japan). The running time to exhaustion was recorded when mice were incapable of keeping running. Exhaustion was defined as the inability to continue regular treadmill running despite the stimulation of repeated tapping on the back of the mouse.

### 2.3. Sampling and Measurements

#### 2.3.1. Sample Processing

Mice in the Ex and GE + Ex groups were sacrificed immediately after exercise with the inhaling with isoflurane (Abbott, Tokyo, Japan), or at the same average time after orally administering (Con and GE groups). When mice were under anesthesia, blood samples were collected from the abdominal artery. Then, after hemoperfusion and dislocation of the spine, skeletal muscles (gastrocnemius and soleus) and livers were excised and immediately frozen in liquid nitrogen. Blood samples were centrifuged at 4 °C for 10 min at 1600× *g* to get heparinized plasma.

#### 2.3.2. Measurement of Plasma Biochemical Parameters

The plasma levels of albumin, aspartate aminotransferase (AST), alanine aminotransferase (ALT), lactate dehydrogenase (LDH), creatine kinase (CK), triglyceride, non-esterified fatty acid (NEFA), uric acid (UA), urea nitrogen (BUN), creatinine, and glucose were measured by Kotobiken Medical Laboratories (Ibaraki, Japan). The methods adopted are in accordance with the Japan Society of Clinical Chemistry’s (JSCC) or the International Federation of Clinical Chemistry and Laboratory Medicine’s (IFCC) standardized assay.

#### 2.3.3. Measurement of Oxidative Stress Markers

Thiobarbituric acid reactive substance (TBARS) and protein carbonyl concentrations are frequently used to assess oxidative stress status. Both of these markers were measured in the liver and skeletal muscles using a TBARS Assay Kit (Cayman Chemical Co., Ann Arbor, MI, USA. Item no. 10009055) and Protein Carbonyl Assay Kit (Cayman Chemical Co., Arbor, MI, USA. Item no. 10005020). All analyses were performed with technical duplication.

#### 2.3.4. Real-Time Quantitative Polymerase Chain Reaction (RT-qPCR)

The total RNA of gastrocnemius muscle was extracted using RNeasy Mini Kit (Qiagen, Valencia, CA, USA) in accordance with the instructions. The NanoDrop system was used to measure the purity and concentration of total RNA. Afterwards, total RNA was reverse transcribed to cDNA using the High Capacity cDNA Reverse Transcription Kit (Applied Biosystems, Foster City, CA, USA) according to the instructions. RT-qPCR was performed with the Fast 7500 real-time PCR system (Applied Biosystems, Foster City, CA, USA) using TaqMan™ Gene Expression Master Mix (Applied Biosystems, Foster City, CA, USA. Item no. 4369016). The thermal profiles for all measured genes composed by 10 min of denaturation at 95 °C, 40 cycles of denaturing at 95 °C for 3 s, and annealing at 60 °C for 15 s. Moreover, 18S mRNA was used in this experiment as the housekeeping gene. Target gene expression levels were quantified using the ΔΔCT method and showed as fold change relative to the values of the Con group. Primer sequences has been listed below (Table 1).

#### 2.3.5. Data Analysis

A two-way analysis of variance (ANOVA) was performed to identify the main effects of GE and/or exercise. “Interaction” means interaction of GE and exercise. When results showed significant interaction, a Bonferroni post-hoc test was performed to identify differences among groups. Data are presented as mean ± SE.

## 3. Results

### 3.1. Effect of Single-Dose GE Administration on Endurance Running Capacity

The running time to exhaustion and the average total running distance of the Ex group (181 ± 15 min, a total running distance of 3939 m, *n* = 8), and GE + Ex group (177 ± 15 min, 3843 m, *n* = 8) did not show significant differences (*p* > 0.05).

### 3.2. Effect of Exhaustive Exercise and GE Administration on Blood Biomarkers

Glucose level significantly decreased in the exercise group because glucose was utilized as a source of energy. NEFA and BUN concentrations increased immediately after exhaustive exercise. Moreover, the muscle damage markers CK and LDH levels increased after exercise, along with the liver or muscle damage markers AST and ALT. However, all of the organ damage markers were not converted by GE administration (Table 2).

### 3.3. Effects of Exhaustive Exercise and GE Administration on Inflammation-Related Gene Expression Levels of Skeletal Muscle

**Figure 2:**
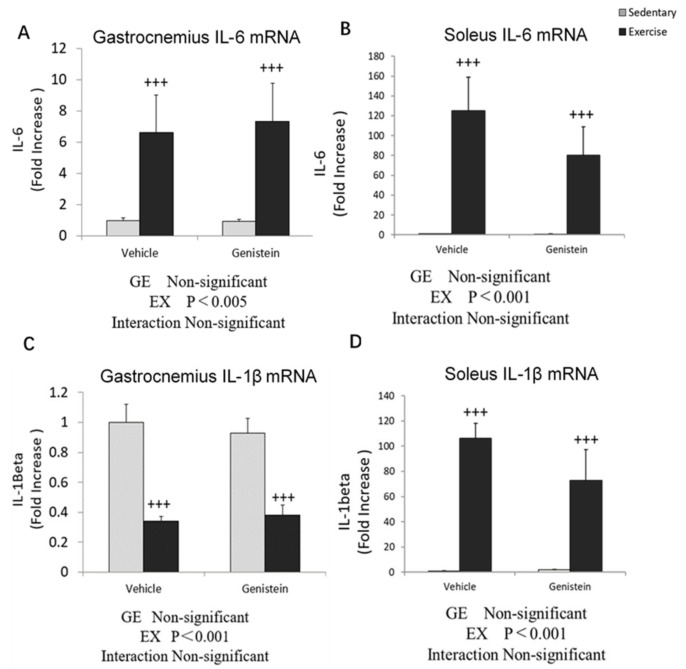
(**A**) Gastrocnemius IL-6 mRNA (Con, *n* = 8; GE, *n* = 8; Ex, *n* = 8; GE+EX, *n* = 8). (**B**) Soleus IL-6 mRNA (Con, *n* = 8; GE, *n* = 8; Ex, *n* = 8; GE + EX, *n* = 8). (**C**) Gastrocnemius IL-1β mRNA (Con, *n* = 7; GE, *n* = 7; Ex, *n* = 7; GE+EX, *n* = 7). (**D**) Soleus IL-1β mRNA (Con, *n* = 7; GE, *n* = 7; Ex, *n* = 7; GE+EX, *n* = 7). Vehicle: non-supplemented groups (only administrated with 0.5% CMC-Na solution), GE: GE-supplemented groups. +++ *p* < 0.001, the main effect of exercise. Values are means ± SE.

The gene expression levels of inflammation-related cytokine *IL-6* and *IL-1β* were measured to identify the effects of GE administration in gastrocnemius and soleus. The results are presented as the relative differences compared with the Con group. In gastrocnemius, exhaustive exercise increased the *IL-6* mRNA expression level by 6.6-fold (Figure 2A), but suppressed the expression of *IL-1β* (Figure 2C). Exhaustive exercise robustly increased the *IL-6* and *IL-1β* mRNA expression levels in soleus by nearly 120-fold and 100-fold (Figure 2B,D). However, no significant differences in the expression levels of those cytokines were induced by GE treatment.

### 3.4. Markers of Oxidative Stress in Skeletal Muscles

**Figure 3:**
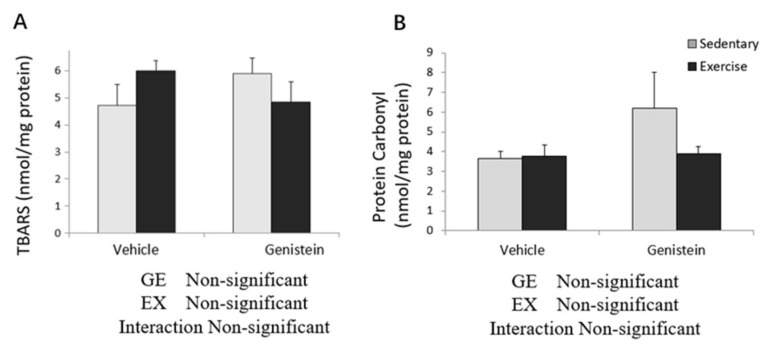
(**A**) Skeletal muscle TBARS (Con, *n* = 6; GE, *n* = 7; Ex, *n* = 8; GE + EX, *n* = 6); (**B**) protein carbonyl (Con, *n* = 5; GE, *n* = 7; Ex, *n* = 8; GE + EX, *n* = 6), vehicle: non-supplemented groups (only administrated with 0.5% CMC-Na solution); GE: GE-supplemented groups. Values are means ± SE.

Concentrations of skeletal muscle TBARS and protein carbonyls were not significantly changed by exercise or GE treatment (Figure 3A,B).

### 3.5. Effects of Exhaustive Exercise and GE Administration on Antioxidant Capacity-Related Gene Expression Levels of Skeletal Muscle

**Figure 4:**
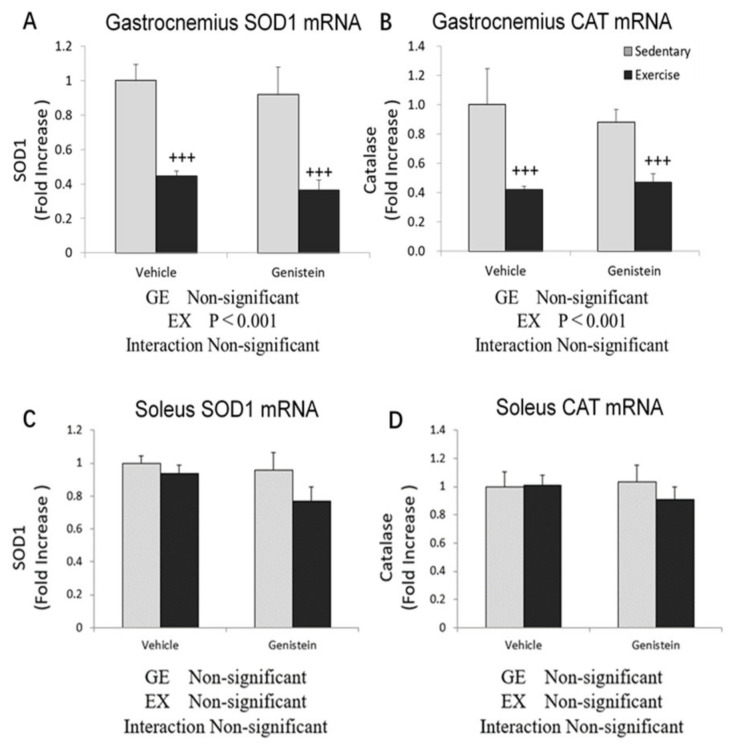
(**A**) Gastrocnemius *SOD1* mRNA (Con, *n* = 7; GE, *n* = 8; Ex, *n* = 8; GE + EX, *n* = 8). (**B**) Gastrocnemius *CAT* mRNA (Con, *n* = 7; GE, *n* = 8; Ex, *n* = 8; GE + EX, *n* = 8). (**C**) Soleus *SOD1* mRNA (Con, *n* = 7; GE, *n* = 7; Ex, *n* = 7; GE + EX, *n* = 7). (**D**) Soleus *CAT* mRNA (Con, *n* = 7; GE, *n* = 7; Ex, *n* = 6; GE + EX, *n* = 7). Vehicle: non-supplemented groups (only administrated with 0.5% CMC-Na solution). GE: GE-supplemented groups. +++ *p* < 0.001, the main effect of exercise. Values are means ± SE.

Significantly decreased superoxide dismutase 1 (*SOD1*) and *CAT* gene expression was observed between the sedentary group and exhaustive exercise group in gastrocnemius. However, no effect of GE administration was observed in the present experiment. In soleus, no significant differences of *SOD1* and *CAT* gene expression levels were observed between each group (Figure 4A,B).

### 3.6. Effect of Exhaustive Exercise and GE Administration on Nrf2 and HO-1 Expression Level of Skeletal Muscle

**Figure 5:**
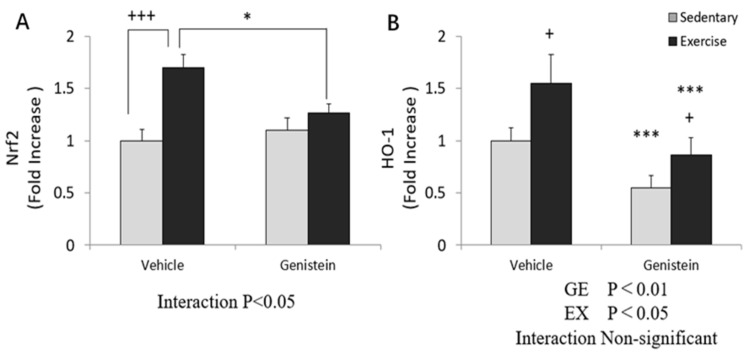
(**A**) Nrf2 mRNA (Con, *n* = 8; GE, *n* = 8; Ex, *n* = 8; GE + EX, *n* = 8); (**B**) HO-1 mRNA (Con, *n* = 6; GE, *n* = 8; Ex, *n* = 8; GE + EX, *n* = 8), vehicle: non-supplemented groups (only administrated with 0.5% CMC-Na solution); GE: GE-supplemented groups. + *p* < 0.05, +++ *p* < 0.001, the main effect of exercise, * *p* < 0.05, the main effect for GE, *** *p* < 0.001, the main effect for GE. Values are means ± SE.

In gastrocnemius, the gene expression of *Nrf2* showed a significant increase in the Ex group compared with the Con group, and was decreased with GE administration in the exercise group (Figure 5A). On the other hand, *HO-1* gene expression level was increased by exhaustive exercise, but suppressed by the GE treatment (Figure 5B).

**Figure 6:**
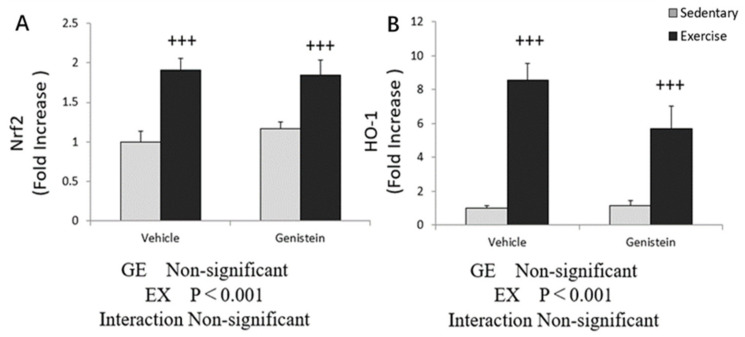
(**A**) *Nrf2* mRNA (Con, *n* = 7; GE, *n* = 7; Ex, *n* = 7; GE + EX, *n* = 7); (**B**) *HO-1* mRNA (Con, *n* = 7; GE, *n* = 7; Ex, *n* = 7; GE + EX, *n* = 7), vehicle: non-supplemented groups (only administrated with 0.5% CMC-Na solution); GE: GE-supplemented groups. +++ *p* < 0.001, the main effect of exercise. Values are means ± SE.

In soleus, the gene expression of *HO-1* and *Nrf-2* showed a significant increase in the Ex groups compared with Con groups (Figure 6A,B), and *HO-1* gene expression was slightly decreased with GE administration in the exercise group (Figure 6B).

### 3.7. Effects of Exhaustive Exercise and GE Administration on Inflammation-Related Gene Expression Levels of Liver

**Figure 7:**
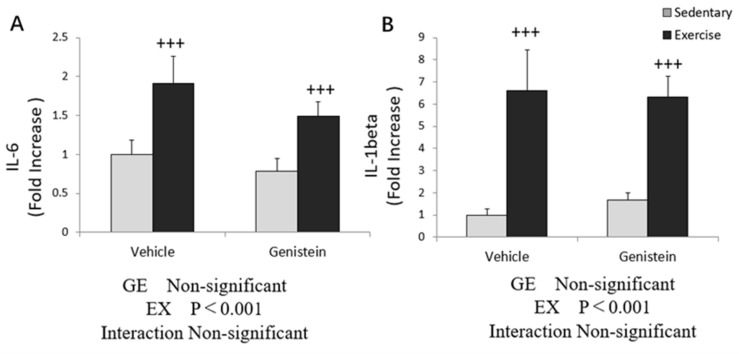
(**A**) *IL-6* mRNA (Con, *n* = 7; GE, *n* = 7; Ex, *n* = 7; GE+EX, *n* = 7); (**B**) *IL-1β* mRNA (Con, *n* = 8; GE, *n* = 7; Ex, *n* = 6; GE+EX, *n* = 7), vehicle: non-supplemented groups (only administrated with 0.5% CMC-Na solution); GE: GE-supplemented groups. +++ *p* < 0.001, the main effect of exercise. Values are means ± SE.

Exhaustive exercise increased the *IL-6* and *IL-1β* mRNA expression levels in soleus, but no effect of GE administration was observed (Figure 7A,B).

### 3.8. Markers of Oxidative Stress in Liver

**Figure 8:**
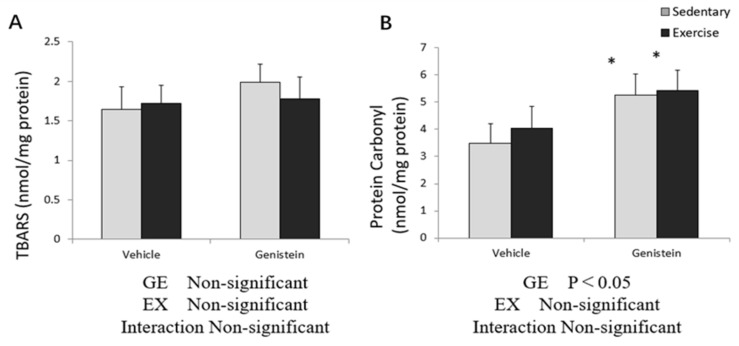
(**A**) Liver TBARS (Con, *n* = 7; GE, *n* = 7; Ex, *n* = 8; GE + EX, *n* = 8); (**B**) protein carbonyl (Con, *n* = 8; GE, *n* = 7; Ex, *n* = 8; GE + EX, *n* = 8), vehicle: non-supplemented groups (only administrated with 0.5% CMC-Na solution); GE: GE-supplemented groups. * *p* < 0.05, main effect for GE treatment. Values are means ± SE.

Concentration of liver TBARS showed no significant difference in the exercise or GE treatment (Figure 8A). Liver protein carbonyl concentrations increased significantly after GE treatment, in both the sedentary group and exercise group (Figure 8B).

### 3.9. Effects of Exhaustive Exercise and GE Administration on Antioxidant Capacity-Related Gene Expression Levels of Liver

**Figure 9:**
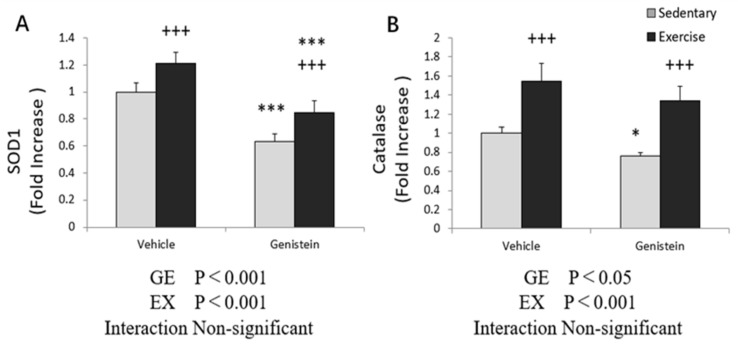
(**A**) *SOD1* mRNA (Con, *n* = 7; GE, *n* = 7; Ex, *n* = 7; GE + EX, *n* = 7); (**B**) *CAT* mRNA (Con, *n* = 8; GE, *n* = 7; Ex, *n* = 6; GE + EX, *n* = 7), vehicle: non-supplemented groups (only administrated with 0.5% CMC-Na solution); GE: GE-supplemented groups. +++ *p* < 0.001, the main effect of exercise. * *p* < 0.05, *** *p* < 0.001 main effect for GE treatment. Values are means ± SE.

Significantly increased *SOD1* and *CAT* gene expression was observed between the sedentary group and exhaustive exercise group. However, a suppressive effect of GE administration was observed irrespective of exercise in the present experiment (Figure 9A,B).

### 3.10. Effects of Exhaustive Exercise and GE Administration on Nrf2 and HO-1 Expression Levels of Liver

**Figure 10:**
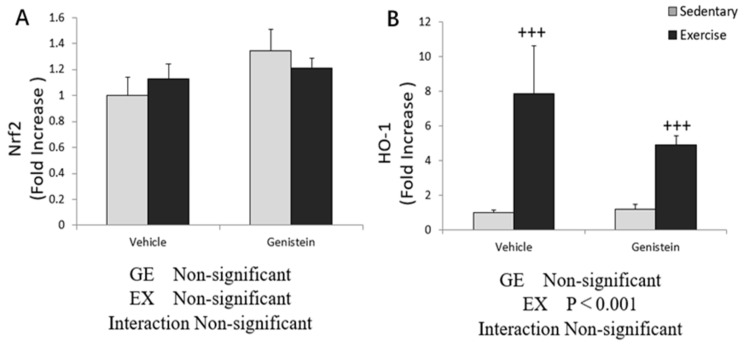
(**A**) *Nrf*2 mRNA (Con, *n* = 8; GE, *n* = 7; Ex, *n* = 7; GE + EX, *n* = 7); (**B**) *HO-1* mRNA (Con, *n* = 8; GE, *n* = 7; Ex, *n* = 7; GE + EX, *n* = 7), vehicle: non-supplemented groups (only administrated with 0.5% CMC-Na solution); GE: GE-supplemented groups. +++ *p* < 0.001, the main effect of exercise. Values are means ± SE.

The gene expression of *HO-1* showed a significant increase in the Ex group compared with the Con group, and was slightly decreased with GE administration in the exercise group (Figure 10B).

## 4. Discussion

In this study, we investigated the effects of a single, high-dose GE supplementation on exhaustive exercise-induced inflammation and oxidative stress in mouse skeletal muscle and liver. As the main finding of this study, we revealed that GE administration failed to relieve oxidative stress, but increased oxidative stress in the liver, which is shown by the increase of protein carbonyl, and might be associated with the failure to regulate *Nrf2* gene expression.

Intensive physical activity, different from mild-intensity physical exercise, triggers inflammation and oxidative stress in different tissues, which may be a result of increased oxygen consumption during intensive muscle contraction [19]. In line with previous studies, we found that exhaustive exercise increased ALT and AST levels, reflecting liver or muscle damage. On the other hand, some of the inflammation-related gene expression levels, such as *IL-6* and *IL-1β,* increased in the liver and skeletal muscles after exercise. However, in this study, we did not observe that GE supplementation attenuated the liver damage or inflammation, as reported by a previous study [20]. Moreover, it is worth mentioning that the *IL-6* gene expression level remarkably increased by 120-fold in the slow-twitch muscles after exercise in the present study. This notably increase of *IL-6* gene expression level was not found in the fast-twitch muscle. However, the difference between the different types of muscle fibers might be attributed to a differences in the basal status. To clarify whether the absolute production of IL-6 differs in the two types of muscle fibers, further studies are encouraged.

It has been widely proved that GE, as the representative member of the isoflavonoids family, could act as a potent antioxidant substance because of its phytoestrogen function [21]. GE can alleviate oxidative stress caused by exercise, which has been proven to some extent, but there are still studies that have demonstrated GE intake did not ameliorate oxidative stress induced by exercise [22]. It is widely known that GE has a strong effect of increasing antioxidative capacity. To date, there are many studies that have proven that GE induced an increase in the activity of the endogenous antioxidant system, such as the increase in the activity of SOD [23], glutathione peroxidase (GPx), and glutathione (GSH) [24].

In this study, we examined TBARS and protein carbonyl concentrations to assess the effect of GE administration on antioxidant capacity and gene expression of *SOD1* and *CAT* to evaluate the effect of GE administration on enzymatic antioxidant capacity. However, contrary to our expectations, we observed an increase of protein carbonyl in the liver. These results revealed that protein oxidation in the liver is more severe after GE treatment. In previous studies, the antioxidant effects of flavonoids supplementation showed inconsistent results, with some showing antioxidant effects and others showing pro-oxidant effects. The mixed results are likely related to long-term administration or high-dose administration [25,26]. Some substances lead to the production of highly reactive compounds that, although they help prevent infection, can also lead to significant inflammation and tissue damage. Some popular and well-known antioxidant flavonoids were reported to also act as pro-oxidants when a transition metal is available, while GE was reported to be an extraordinary ROS scavenger [27]. The present results indicate that a single dose of GE administration may induce pro-oxidant effects in the liver and, therefore, fail to exert its anti-oxidation ability. Consistent with our results, Chen et al. demonstrated that a high concentration of GE (200 µM) stimulated ROS generation, mediated by 5-lipoxygenase. Furthermore, a high concentration of GE elevated lipid peroxidation, which indicates an imbalance in the cellular redox system [25].

Although repeated administration of GE was reported to extensively show antioxidant capacities, such as an estrogenic substance, long-term GE may impair exercise performance in athletes. Here, we investigated whether a single dose of GE, of 200 mg/kg, indicates consequential antioxidant capacity through the antioxidant enzyme system. Contrary to our expectation, a single administration, of a 200 mg/kg oral dose of GE, did not show any influence on *SOD1* and *CAT* gene expression in skeletal muscles. Furthermore, we observed that GE has a suppressive effect on liver *SOD1* and *CAT* gene expression in both the excise group and the sedentary group. Recently, some studies also observed a suppressive function of GE on antioxidative enzymes. Singh et al. administered GE by different concentration ranges, from 125 to 1000 mg/kg; *SOD1* and *CAT* mRNA levels decreased significantly in the high-dose treatment group, but increased in the low-dose treatment group [28]. Röhrdanz et al. observed flavonoid supplementation brought a downregulation of various antioxidant enzymes, such as Cu, Zn-SOD, Mn-SOD, and GPx [24]. We suspected that this downregulation might be related to the inhibition of Nrf2 gene expression, which is caused by high-dose GE treatment.

The activation of the Nrf2/ARE pathway is the main way to stimulate the antioxidant defense system and other cytoprotective genes, such as *HO-1,* which is related to the release of oxidative stress [29]. There are plenty of studies indicating that relatively low-dose GE can activate the Nrf2/ARE pathway to protect the body from oxidative stress and inflammation [30,31]. At the same time, some studies showed flavonoids may act as a dual role in the activation of the Nrf2/ARE pathway. This might be related to the dosage and the duration [32]. For instance, a low exposure concentration of apigenin (6.25 µM) was able to activate the Nrf2/ARE pathway, improving the gene expression level of *Nrf2* and *HO-1* through the activation of ERK1/2 signaling [33]. On the other hand, the high exposure concentration of apigenin decreased the gene expression level and protein concentrations of Nrf2 (100 µM) and CAT activity [34]. Likewise, luteolin showed different effects on the Nrf2/ARE pathway due to exposure concentration. At low concentrations, luteolin activated Nrf2/ARE pathway, but exhibited inhibitory effects at high concentrations [35,36]. In the present study, we found that *Nrf2* and *HO-1* gene expression levels were significantly suppressed in skeletal muscles in GE administration groups. Moreover, *HO-1* expression was slightly decreased in the liver after GE administration. Under the premise of the above-mentioned view, we assumed that, in this study, a single, high-dose of GE would not show any preventive effect on exhaustive exercise-induced oxidative stress, as GE acts as a pro-oxidant by the inhibition activity of Nrf2 and downstream gene expression in the liver; thus, influencing the oxidative stress systematically. Except for the Nrf2 pathway, there are several mechanisms involved in the pro-oxidant effects of genistein. Singh et al. indicated that high concentration of genistein also downregulated several glutathione metabolism-related gene expression levels, such as glutathione s-transferase pi 1 (GSTP1) and microsomal glutathione s-transferase 1 (MGST1) [28]. It is also reported that genistein exerts pro-oxidant potential in the primary muscle cells through enhancing ROS production in a 5-lipoxygenase-dependent manner [25]. Furthermore, previous studies have reported that *HO-1* expression may not be dependent on *Nrf2* activation [37]. The expression of *HO-1* is also regulated by transcription activators or repressors, such as Bach1, a transcription factor [37]. The mechanism that we investigated in the present study is not enough to explain the pro-oxidant effects of genistein. In further research, more upstream stimulatory factors need to be investigated.

Since we aimed to report on the mechanism of the signal transduction, we only measured gene expression of selected cytokines and enzymes. Parameters, such as the ROS, which reflects dynamic oxidative stress, or the GSH/glutathione disulfide (GSSG) ratio, an increased ratio of which is an indication of oxidative stress should be measured in the further studies. Although there are multiple published data on long-term, low doses of GE administration regulating phase 2 detoxification and antioxidant enzyme expression, very little is known about the short-term, high doses of GE on exercise-induced oxidation and inflammation. According to our findings, further studies are encouraged to use relatively low-dose and long-term GE supplementation to elicit its health-promoting effects. Furthermore, isoflavonoids show estrogenic effects in organisms; therefore, administration in female animals may present interesting results due to gender difference, and further study is encouraged.

## 5. Conclusions

In conclusion, the findings of the present studies are: (1) acute exercise was able to induce organ damage, inflammation, and oxidative stress in the skeletal muscles and liver. (2) A single dose of 200 mg/kg body weight of GE supplementation before exercise did not lead to favorable antioxidant and anti-inflammatory effects in this study, at least for the parameters we investigated. (3) Oxidative stress in the liver was actually slightly induced by GE supplementation, along with the suppression of antioxidant enzyme expression. Further studies are encouraged to discover the optimal administration dosage and method of GE to obtain maximum benefits in the sports science field.

## Figures and Tables

**Table 1:** Sequences of primer pairs used in sequences of primer pairs used in reverse transcription polymerase chain reaction.

Target Gene	Forward	Reverse
*18S*	CGGCTACCACATCCAAGGA	AGCTGGAATTACCGCGGC
*IL-6*	TAGTCCTTCCTACCCCAATTTCC	TTGGTCCTTAGCCACTCCTTC
*IL-1β*	GGGCCTCAAAGGAAAGAATC	TTGCTTGGGATCCACACTCT
*SOD1*	GAGACCTGGGCAATGTGACT	GTTTACTGCGCAATCCCAAT
*CAT*	CAGAGAGCGGATTCCTGAGAGA	CTTTGCCTTGGAGTATCTGGTGAT
*Nrf2*	GAGTCGCTTGCCCTGGATATC	TCATGGCTGCCTCCAGAGAA
*HO-1*	CACGCATATACCCGCTACCT	CCAGAGTGTTCATTCGAGCA

**Table 2:** The results of blood biomarkers analysis.

Biomarker	Con	GE	Ex	GE + Ex
Glucose (mg/dL)	234.4 ± 11.7	217.9 ± 12.3	172.1 ± 23.8 ^+++^	139.5 ± 17.6 ^+++^
NEFA (μmol/L)	2.2 ± 0.3	2.1 ± 0.3	3.0 ± 0.1 ^+++^	3.2 ± 0.1 ^+++^
BUN (mg/dL)	22.7 ± 1.5	23.9 ± 1.5	35.6 ± 1.7 ^+++^	38.7 ± 2.5 ^+++^
CK (IU/L)	950.6 ± 259.2	806.3 ± 258.0	5358.8 ± 830.4 ^+++^	6585.4 ± 1331.1 ^+++^
LDH (IU/L)	438.4 ± 56.3	406.9 ± 59.6	1690.5 ± 165.2 ^+++^	1716.0 ± 253.3 ^+++^
ALT (IU/L)	32.6 ± 2.0	33.4 ± 3.4	122.6 ± 19.3 ^+++^	119.6 ± 20.1 ^+++^
AST (IU/L)	120.8 ± 14.3	152.6 ± 28.2	500.6 ± 56.3 ^+++^	485.6 ± 66.7 ^+++^

^+++^*p* < 0.001, the main effect of exercise. Values are means ± SE.

## References

[1] Newsholme P., Cruzat V.F., Keane K.N., Carlessi R., de Bittencourt P.I. (2016). Molecular mechanisms of ROS production and oxidative stress in diabetes. Biochem. J..

[2] Suzuki K., Tominaga T., Ruhee R.T., Ma S. (2020). Characterization and modulation of systemic inflammatory response to exhaustive exercise in relation to oxidative stress. Antioxidants.

[3] Penedo F.J., Dahn J.R. (2005). Exercise and well-being: A review of mental and physical health benefits associated with physical activity. Curr. Opin. Psychiatry.

[4] Suzuki K. (2019). Chronic Inflammation as an Immunological Abnormality and Effectiveness of Exercise. Biomolecules.

[5] Suzuki K., Totsuka M., Nakaji S., Yamada M., Kudoh S., Liu Q., Sugawara K., Yamaya K., Sato K. (1999). Endurance exercise causes interaction among stress hormones, cytokines, neutrophil dynamics, and muscle damage. J. Appl. Physiol..

[6] Radak Z., Chung H.Y., Goto S. (2005). Exercise and hormesis: Oxidative stress-related adaptation for successful aging. Biogerontology.

[7] Malaguti M., Angeloni C., Hrelia S. (2013). Polyphenols in exercise performance and prevention of exercise-induced muscle damage. Oxid. Med. Cell Longev..

[8] Tidball J.G. (1995). Inflammatory cell response to acute muscle injury. Med. Sci. Sports Exerc..

[9] Kawanishi N., Mizokami T., Niihara H., Yada K., Suzuki K. (2016). Neutrophil Depletion Attenuates Muscle Injury after Exhaustive Exercise. Med. Sci. Sports Exerc..

[10] Incir S., Bolayirli I.M., Inan O., Aydın M.S., Bilgin I.A., Sayan I., Esrefoglu M., Seven A. (2016). The effects of genistein supplementation on fructose induced insulin resistance, oxidative stress and inflammation. Life Sci..

[11] Ruhee R.T., Ma S., Suzuki K. (2019). Sulforaphane Protects Cells against Lipopolysaccharide-Stimulated Inflammation in Murine Macrophages. Antioxidants.

[12] Ma S., Yada K., Lee H., Fukuda Y., Iida A., Suzuki K. (2017). Taheebo polyphenols attenuate free fatty acid-induced inflammation in murine and human macrophage cell lines as inhibitor of cyclooxygenase-2. Front. Nutr..

[13] Kwon S.H., Kang M.J., Huh J.S., Ha K.W., Lee J.R., Lee S.K., Lee B.S., Han I.H., Lee M.S., Lee M.W. (2007). Comparison of oral bioavailability of genistein and genistin in rats. Int. J. Pharm..

[14] Uruno A., Motohashi H. (2011). The Keap1-Nrf2 system as an in vivo sensor for electrophiles. Nitric Oxide.

[15] Lignitto L., LeBoeuf S.E., Homer H., Jiang S., Askenazi M., Karakousi T.R., Pass H.I., Bhutkar A.J., Tsirigos A., Ueberheide B. (2019). Nrf2 Activation Promotes Lung Cancer Metastasis by Inhibiting the Degradation of Bach1. Cell.

[16] Done A.J., Traustadóttir T. (2016). Nrf2 mediates redox adaptations to exercise. Redox Biol..

[17] Yang L., Shen L., Li Y., Li Y., Yu S., Wang S. (2017). Hyperoside attenuates dextran sulfate sodium-induced colitis in mice possibly via activation of the Nrf2 signalling pathway. J Inflamm..

[18] Supko J.G., Malspeis L. (1995). Plasma pharmacokinetics of genistein in mice. Int. J. Oncol..

[19] Sanchis-Gomar F., Pareja-Galeano H., Perez-Quilis C., Santos-Lozano A., Fiuza-Luces C., Garatachea N., Lippi G., Lucia A. (2015). Effects of allopurinol on exercise-induced muscle damage: New therapeutic approaches?. Cell Stress Chaperones.

[20] Shenoy S., Dhawan M., Singh Sandhu J. (2016). Four Weeks of Supplementation with Isolated Soy Protein Attenuates Exercise-Induced Muscle Damage and Enhances Muscle Recovery in Well Trained Athletes: A Randomized Trial. Asian J. Sports Med..

[21] Rahman Mazumder M.A., Hongsprabhas P. (2016). Genistein as antioxidant and antibrowning agents in in vivo and in vitro: A review. Biomed. Pharmacother..

[22] Chen C.Y., Bakhiet R.M., Hart V., Holtzman G. (2005). Isoflavones improve plasma homocysteine status and antioxidant defense system in healthy young men at rest but do not ameliorate oxidative stress induced by 80% VO2pk exercise. Ann. Nutr. Metab..

[23] Ismail M., Ibrahim S., El-Amir A., El-Rafei A.M., Allam N.K., Abdellatif A. (2018). Genistein Loaded Nanofibers Protect Spinal Cord Tissue Following Experimental Injury in Rats. Biomedicines.

[24] Zhang H., Zhao Z., Pang X., Yang J., Yu H., Zhang Y., Zhou H., Zhao J. (2017). MiR-34a/sirtuin-1/foxo3a is involved in genistein protecting against ox-LDL-induced oxidative damage in HUVECs. Toxicol. Lett..

[25] Chen W., Lin Y.C., Ma X.Y., Jiang Z.Y., Lan S.P. (2014). High concentrations of genistein exhibit pro-oxidant effects in primary muscle cells through mechanisms involving 5-lipoxygenase-mediated production of reactive oxygen species. Food Chem. Toxicol..

[26] Chen W., Ma X., Lin Y., Xiong Y., Zheng C., Hu Y., Yu D., Jiang Z. (2016). Dietary supplementation with a high dose of daidzein enhances the antioxidant capacity in swine muscle but experts pro-oxidant function in liver and fat tissues. J. Anim. Sci. Biotechnol..

[27] Kruk I., Aboul-Enein H.Y., Michalska T., Lichszteld K., Kładna A. (2005). Scavenging of reactive oxygen species by the plant phenols genistein and oleuropein. Luminescence.

[28] Tian H., Zhou G., Zhu Z. (2015). Evaluation of Cardioprotective Effects of Genistein against Diabetes-induced Cardiac Dysfunction in Rats. Trop. J. Pharm. Res..

[29] Singh P., Sharma S., Rath S.K. (2014). Genistein induces deleterious effects during its acute exposure in Swiss mice. BioMed Res. Int..

[30] Ruhee R.T., Ma S., Suzuki K. (2020). Protective Effects of Sulforaphane on Exercise-Induced Organ Damage via Inducing Antioxidant Defense Responses. Antioxidants.

[31] Miao Z.Y., Xia X., Che L., Song Y.T. (2018). Genistein attenuates brain damage induced by transient cerebral ischemia through up-regulation of Nrf2 expression in ovariectomized rats. Neurol. Res..

[32] Liu F.C., Wang C.C., Lu J.W., Lee C.H., Chen S.C., Ho Y.J., Peng Y.J. (2019). Chondroprotective Effects of Genistein against Osteoarthritis Induced Joint Inflammation. Nutrients.

[33] Suraweera T.L., Rupasinghe H., Dellaire G., Xu Z. (2020). Regulation of Nrf2/ARE Pathway by Dietary Flavonoids: A Friend or Foe for Cancer Management?. Antioxidants.

[34] Paredes-Gonzalez X., Fuentes F., Jeffery S., Saw C.L., Shu L., Su Z.Y., Kong A.N. (2015). Induction of NRF2-mediated gene expression by dietary phytochemical flavones apigenin and luteolin. Biopharm. Drug. Dispos..

[35] Valdameri G., Trombetta-Lima M., Worfel P.R., Pires A.R., Martinez G.R., Noleto G.R., Cadena S.M., Sogayar M.C., Winnischofer S.M., Rocha M.E. (2011). Involvement of catalase in the apoptotic mechanism induced by apigenin in HepG2 human hepatoma cells. Chem. Biol. Interact..

[36] Yang Y., Cai X., Yang J., Sun X., Hu C., Yan Z., Xu X., Lu W., Wang X., Cao P. (2014). Chemoprevention of dietary digitoflavone on colitis-associated colon tumorigenesis through inducing Nrf2 signaling pathway and inhibition of inflammation. Mol. Cancer.

[37] Drummond G.S., Baum J., Greenberg M., Lewis D., Abraham N.G. (2019). HO-1 overexpression and underexpression: Clinical implications. Arch. Biochem..

